# The impact of commercial health datasets on medical research and health-care algorithms

**DOI:** 10.1016/S2589-7500(23)00025-0

**Published:** 2023-05

**Authors:** Isabelle Rose I Alberto, Nicole Rose I Alberto, Arnab K Ghosh, Bhav Jain, Shruti Jayakumar, Nicole Martinez-Martin, Ned McCague, Dana Moukheiber, Lama Moukheiber, Mira Moukheiber, Sulaiman Moukheiber, Antonio Yaghy, Andrew Zhang, Leo Anthony Celi

**Affiliations:** College of Medicine, University of the Philippines, Manila, Philippines (I R I Alberto BS, N R I Alberto BS); Department of Medicine, Weill Cornell Medical College, Cornell University, New York, NY, USA (A K Ghosh MD MSc); Institute for Medical Engineering and Science (B Jain BS, N McCague MPH, D Moukheiber MS, L Moukheiber MS, A Yaghy MD, A Zhang AB, L A Celi MD MS) and The Picower Institute for Learning and Memory (M Moukheiber MS), Massachusetts Institute of Technology, Cambridge, MA, USA; Deloitte Consulting, London, UK (S Jayakumar MBBS); Stanford Center for Biomedical Ethics, Stanford Medicine, Stanford, CA, USA (N Martinez-Martin JD PhD); Markforged, Watertown, MA, USA (N McCague); Department of Computer Science, Worcester Polytechnic Institute, Worcester, MA, USA (S Moukheiber BS); New England Eye Center, Tufts University Medical Center, Boston, MA, USA (A Yaghy); Department of Stem Cell and Regenerative Biology, Harvard University, Cambridge, MA, USA (A Zhang AB); Department of Medicine, Beth Israel Deaconess Medical Center, Boston, MA, USA (L A Celi); Department of Biostatistics, Harvard T H Chan School of Public Health, Boston, MA, USA (L A Celi)

## Abstract

As the health-care industry emerges into a new era of digital health driven by cloud data storage, distributed computing, and machine learning, health-care data have become a premium commodity with value for private and public entities. Current frameworks of health data collection and distribution, whether from industry, academia, or government institutions, are imperfect and do not allow researchers to leverage the full potential of downstream analytical efforts. In this Health Policy paper, we review the current landscape of commercial health data vendors, with special emphasis on the sources of their data, challenges associated with data reproducibility and generalisability, and ethical considerations for data vending. We argue for sustainable approaches to curating open-source health data to enable global populations to be included in the biomedical research community. However, to fully implement these approaches, key stakeholders should come together to make health-care datasets increasingly accessible, inclusive, and representative, while balancing the privacy and rights of individuals whose data are being collected.

## Introduction

With the increasing digitisation of medical records, the amount of health data produced by medical institutions has grown exponentially.^1,2^ Coupled with the advent of cloud data storage, distributed computing, and machine learning, we have entered a new, digital-first era in medicine that has the potential to use huge volumes of health-care data to accelerate scientific discovery, improve health-care quality, enable personalised medicine, and inform evidence-based policy making.^1,3–5^ We have also seen an increase in researchers working to combine multiple data sources to identify vulnerable groups, quantify inequities in care, and explore the effect of the social determinants of health.^6,7^

This confluence of factors has turned health-care data—including patient demographics, clinical examination results, laboratory findings, and genomic data—into a premium commodity possessing value for private and public entities. The need for large amounts of health-care data in both academia and private industry has brought about an aggressive and lucrative push towards the commercialisation, spawning a multibillion US$ industry that centres on collecting, analysing, and selling these data.^2,6,8–10^

Although essential to the world of drug development and medical devices, these commercially available datasets might be improperly used by academic researchers who are probably accustomed to publicly available datasets (eg, claims data from the US Centers for Medicare and Medicaid Services^11^) and local datasets from their own institutions and studies. The implications of this knowledge and experience gap are non-trivial—academic research directly influences the development of health-care tools, decisions, and policies that have consequences for patients. Commercial datasets might warrant special caution regarding their implications for research reproducibility and generalisability because these datasets are often expensive to access, and thus are access-restricted. Clinicians, patients, researchers, and policy makers need to understand the broader market for biomedical data, including how the datasets are processed and curated, to better inform how they might be used. In this Health Policy paper, we highlight the need for caution in the use of these data in the growing field of health algorithm development, especially when artificial intelligence (AI) or machine learning is involved.

## Sources of commercial health data

There are many sources from which data and specimens used for secondary research are procured.^8^ These include patient registries, health-care databases including electronic health records (EHRs), pharmacy and health insurance databases, social media, and patient-powered research networks.^12^ The data from these sources are then deidentified in accordance with Health Insurance Portability and Accountability Act (HIPAA) standards, and can be sold thereafter without public transparency or patient consent.^9^

The commercialisation of deidentified data is not restricted to covered entities, like physicians or medical institutions, as their business associates can also partake in the deidentification and sale of health-care data, as long as their contractual agreement expressly allows them to do so.^9^ These practices have resulted in many commercial health datasets becoming available to researchers and companies (panel). However, most biomedical research these commercial datasets enable is beyond the original routine clinical care and primary research applications the data and biospecimens were collected for.^8^ Patient-centred and business-centred applications of health data are closely linked,^13^ and therefore, several commercial health datasets are marketed to private industry for industry-related purposes.^14,15^ Due to the opaque nature of health dataset curation and data harmonisation, researchers might not be aware of the sources of bias derived from commercial datasets, nor how best to address these sources of bias and associated limitations with their analyses. These datasets differ from open data sources (eg, Medicare and Medicaid claims), for which decisions regarding curation are made publicly available and have been extensively examined for bias.^16^ Such practices mean that commercial health datasets might not be ideally suited for all research applications; researchers using these datasets for academic purposes should be aware of these limitations. The growing use of such datasets in peer-reviewed research might lead to biased results if not evaluated systematically.^8^ Therefore, researchers should become familiar with the strengths and limitations of both commercial and non-commercial datasets, to reduce the risk of obtaining incorrect or biased results.

## Effect of health data vending on biomedical research: challenges with reproducibility and generalisability

The introduction of commercial health datasets into biomedical research has compounded the problem of reproducibility—a key tenet of rigorous scientific research. Reproducibility is often impeded by the unavailability of the original dataset and code by which the data are processed. Also, commercial databases generally restrict access to data that are essential to reproducing published results.^17^ As a result, these financial barriers cause additional hardships that make the pursuit of scientific inquiry structurally inequitable for equally qualified researchers without the necessary financial resources to obtain these datasets. Restricted-access datasets also pose challenges for routine reproducibility audits when journals evaluate studies before publication.^18^ For example, in a study designed to validate a prediction model of inflammatory bowel disease, the authors noted that the original model was developed with US Veterans Health Administration data, which are inaccessible to those outside the Veterans Affairs system.^19,20^ When choosing an alternative dataset to validate the model, the authors of the validation study also used a commercial dataset (Optum EHR), which they note is unavailable to the public due to data licensing agreements.^21^ As a result, just as with the original model, researchers wishing to examine the validation study must license the data themselves.

There are also inherent limitations in non-public health datasets that hinder the generalisability of health research to a broader population. Proprietary models have been shown to decline in performance over time, but are difficult to assess owing to the scarcity of publicly available data.^22^ As an example involving a proprietary health algorithm developed on non-public data, a commonly used sepsis prediction model based on data from Epic EHRs was found to generalise poorly to an external validation set.^23^ The Epic Sepsis Model was implemented at hundreds of US hospitals and was found to have poor discrimination and calibration in predicting sepsis onset.^23^ The widespread use of this model was not based on thorough independent validation, but rather can be attributed to its ease of integration into existing hospital EHR systems produced by Epic.^23^ The data used to produce the model consisted of 405 000 patient encounters from 2013 to 2015, across three hospital systems, making the model susceptible to data drift and other issues in the absence of regular external validation.

Data availability is a crucial issue in machine learning for health research. Due to the complexity and opaque nature of machine learning for health algorithms such as deep learning, it is particularly important for datasets used in machine learning for health research to be tested for bias. Datasets that are homogeneous are likely to yield poorly generalisable models.^24^ Moreover, machine learning algorithms can exploit patterns in the training data that can be imperceptible to a human observer but have the potential to negatively influence care delivery.^25^ Therefore, third parties must evaluate not only machine learning for health models but also the data they are trained on to establish the suitability of these data and model for a particular application. Despite these concerns, only about 55% of machine learning for health studies use publicly available data—a substantially lower percentage than in machine learning studies related to non-health care.^26^ For example, a 2020 study published by Google showing AI application for breast cancer screening^27^ was met with criticism for not releasing model architecture parameters and for using proprietary datasets. The authors of the letter accuse Google of the “promotion of a closed technology”.^28^ Although in this case access to the codebase was also pointed out as an issue, the point remains that restricted-access datasets have made reproducibility and generalisability in machine learning for health particularly challenging.

The repurposing of data from commercial insurance claims for building health-care models can also be problematic. These claims data, which serve as data sources for many commercial databases, are restricted in scope and are subject to upcoding.^29^ In some circumstances, prediction models directly created from medical records are more accurate than models created from claims data possibly because clinical characteristics can be better defined in medical records.^30^ Models for predicting the severity of rheumatoid arthritis solely built with claims data, have been shown to exhibit low accuracy among patients with high disease activity.^31^ Without the purchase of supplemental databases, many key demographic and outcome details such as smoking status, race, and mortality are inaccessible, thereby limiting the scope and extent of research that can be done with these core claims datasets.^32^ Moreover, many claims databases represent working-age patients covered by private insurance supplied by employers rather than a random sample from the US population. For example, employee health data from large employers are used more frequently in databases than data from small or medium firms. Therefore, although these databases cover a large portion of the US population, they might not capture some groups and thereby yield problematic algorithms.

There is also poor consistency in the structure of the underlying data and definition of data across claims of data providers.^33^ Interdatabase discrepancies involving data availability, patient populations, and other characteristics might give rise to inconsistent research results. For example, in a study that assessed the risk of sudden cardiac arrest and ventricular arrhythmia among users of second-generation sulfonylureas, inconsistent results were found with claims from five states’ Medicaid claims (1999–2012) and Optum Clinformatics commercial claims (2000–16).^34^ These discrepancies were partly attributable to differential data capture across insurance plans, data features being present in one dataset but not the other, and demographic differences between the two patient populations (ie, Optum capturing data only from commercially insured individuals; and Medicaid capturing data from publicly insured adults with low-income, older people [≥65 years old], children [<19 years old], pregnant people, and people with disabilities).^34^ The presence of these discrepancies raises issues regarding the breadth of research done with a single commercial dataset, meaning that researchers might be pressured to purchase multiple datasets. Therefore, the suitability of claims data for biomedical research should be carefully evaluated on a case-by-case basis per standardised database selection frameworks. For example, international societies such as the International Society of Pharmaceutical Engineering and International Society for Pharmacoeconomics and Outcomes Research have developed guidelines regarding the analysis of secondary data sources for treatment effectiveness research.^35,36^

## Ethical considerations in data vending

Discussions of the ethics of big data projects in medicine often focus on privacy and data protection, particularly in terms of the risks to individuals from their personal health data being revealed or used for non-medical purposes. HIPAA protects personal health information that is identifiable, but does not place restrictions on the use of deidentified health data to support potential gains in scientific knowledge enabled by sharing data. Furthermore, HIPAA is limited to data that are generated directly by health-care providers or business associates (when an agreement is in place). HIPAA might also be bypassed if patients sign waivers when obtaining medical care or other benefits, or as a condition of employment. Together with the fact that advances in technology and accessibility of large public databases have allowed the re-identification of data with increasing ease, concern has grown over the limitations of current regulations for protecting privacy in health data at the individual and group levels.^2,37^

However, privacy is a concept that is context-dependent and often should be considered in trade-off with other societal rights and values. Privacy provisions for health data are intended to balance privacy with the social good that might come from sharing data.^38^ For big data projects in medicine, efforts to address privacy are also often viewed in terms of trade-offs with the need to support innovation and scientific knowledge.^39^ For example, some intrusions into privacy are considered permissible if they serve a public health or safety purpose.^40^ Individuals report greater willingness to share genomic and health data if these data are used for social benefit.^41^ For this reason, assessments of the ethics of big data health projects need to include careful consideration of the public benefit and scientific knowledge gained from such projects in the larger discussion of privacy.

The consent process is an essential part of informing the people from whom data are being collected of privacy and data protection issues. In primary research, the informed consent process is used to notify patients about privacy issues, such as the type and scope of data collection and potential downstream risks from the data. Traditionally, informed consent takes place once for a single study. Single-instance consent, however, might not convey the range of potential privacy implications and downstream uses, which might include repurposing or aggregation of data; considering the complexities of future inferential analyses that are not yet developed; and the downstream effects of their data, such as use by third parties to determine insurance or mortgage rates.^42^ Furthermore, when health data are collected through consumer devices, informed consent might not apply, and instead terms and conditions, which are often long, dense, and difficult to read, are the main means for informing consumers of data collection.^43^ Nonetheless, much of the justification for proceeding with big data projects rests on the potential social benefits and medical knowledge developed from projects that aggregate and reuse personal data.

Large-scale data collection projects in health-care institutions, such as learning health systems, have prompted efforts to formulate appropriate means to balance privacy and innovation through responsible data governance or stewardship.^44^ Such approaches include minimising risks to individuals and groups from uses of their data, and mechanisms for generating input from stakeholders on data collection and use. For these frameworks, use of personal data to produce scientific gains and social benefit is prioritised. As commercial datasets play an increasing role in health research, it becomes imperative to examine the extent to which they contribute to scientific knowledge and the public good. Leveraging key data governance principles, including regularly consulting marginalised populations and indigenous communities regarding the collection and processing of personal health data, applying best practices in data deidentification to protect patient data privacy and reduce breach risks, and periodically revisiting governance mechanisms as new technologies are introduced through engagement with the aforementioned stakeholders, all represent potential means through which societal benefits can be maximised from health data.^45^

## Discussion

Clive Humby, a British mathematician, commented that “data is the new oil” in 2006.^46^ However, most people neglect the important addendum in the second part of his quip: “[oil is] valuable, but if unrefined it cannot really be used. It has to be changed into gas, plastic, chemicals, etc., to create a valuable entity that drives profitable activity; so must data be broken down, analyzed for it to have value.”^46^ This phrase proved to be prescient. Multibillion dollar tech companies such as Google, Microsoft, Amazon, and Facebook have collected petabytes of data to power and improve their products.^47^ In health care, Google has partnered with Meditech, an EHR company, and Oracle has purchased Cerner, an EHR company secondary only to Epic.^48,49^ Despite massive troves of data existing in the health-care realm, for them to be useful, they must first be aggregated, processed, and curated. Consequently, health-care databases are a necessity for researchers to develop tools that will improve the quality of care.

With the many growing needs for health data, AI-based and machine learning-driven solutions will form the backbone of precision medicine. Currently, such solutions are largely informed by commercially available datasets created from high-income countries comprised of higher-income populations living near hospitals and with lower barriers to care. By contrast, lower-income countries have fewer capabilities to generate and refine data, a process that often requires advanced infrastructure and expertise. Therefore, a large swath of the global population is excluded from most health-care research and innovations. Ibrahim and colleagues^50^ introduced the term data poverty, which they defined as “the inability for individuals, groups, or populations to benefit from a discovery or innovation due to insufficient data that are adequately representative”. Data poverty might have major downstream effects on the quantity and quality of AI-based health-care tools, meaning lower-income populations are less likely to benefit from the rapid technological advances in clinical care.

The task of curating health-care datasets is costly, and a sustainable solution is required to produce them. Commercialisation is one such strategy, but alternative approaches are also being pursued in a range of initiatives from academic and government institutions. With research funding, cooperation of health-care institutions, and support of patients, several groups have created open-access health-care databases. For example, the Massachusetts Institute of Technology Laboratory for Computational Physiology (MIT LCP; Cambridge, MA, USA) has constructed a critical care database called the Medical Information Mart for Intensive Care (MIMIC).^51^ In contrast to commercially available datasets, access to MIMIC only requires users to confirm their identity, complete human research training, and sign a data use agreement. MIMIC’s code repository provides a forum for public discussion and code sharing, promoting transparency, reproducibility, and collaboration between research groups. Now in its fourth iteration, MIMIC has enabled more than 4000 studies that leverage electronic health record data from a single, large US hospital.^52^ Although MIMIC has proved valuable for research studies, the fact that the data are restricted to a single US hospital limits their value for research. How can the success of MIMIC be reproduced on a grand scale?

Other open-access databases have emerged worldwide, including the AmsterdamUMCdb,^53^ the eICU-CRD,^54^ and the High Time-Resolution Intensive Care Unit Dataset.^55^ As an example of a large, publicly available, multi-institutional EHR dataset, eICU-CRD contains data collected from 208 hospitals across the USA and made possible through a collaboration between Philips Healthcare and MIT LCP.^54^ As the value of publicly available research data is increasingly recognised, the US National Institutes of Health (NIH) in October, 2020, announced a new Data Management and Sharing policy, which requires NIH-funded researchers to share their data publicly.^56^ These examples show that an open-access approach to health-care data is a feasible route alongside commercial endeavours. However, like commercial datasets, current open-access datasets are not without issues. They do not necessarily mitigate the problem of generalisability, because many are derived from just one or a few institutions. Truly comprehensive coverage of the global population would require datasets from diverse geographical regions, necessitating a standardised format for data harmonisation. Industry–academia collaborations, convened through partnerships of commercial entities and clinician scientists, are a promising avenue for the creation of large datasets similar to eICU-CRD. The datasets resulting from these collaborations represent a potential solution to the generalisability concerns that relate to data accessibility as outlined. To facilitate these multi-institutional data pipelines, initiatives such as the Observational Health Data Sciences and Informatics programme^57^ and the US Food and Drug Administration’s Sentinel system^58^ have developed guidelines for data quality and standardisation, and software that enables federated data analytics.

There are opportunities to parallel these successes with commercial datasets. To ensure that inherent biases of commercial datasets are well understood, so that researchers can understand the limitations of them, we have two recommendations. First, data providers should provide detailed documentation of the processes and algorithms used to curate and harmonise the data. This provision would allow researchers to develop a full understanding of the data they are working with and anticipate any potential issues that might arise. Second, commercial datasets should be made open to independent third-party assessors to analyse the limitations, validity, and potential issues with their data curation and harmonisation processes. Ideally, this analysis would be done by the researchers themselves, with access to the code and data, who can then interrogate these processes to quantify and describe any sources of bias.

As patients entrust institutions with their health data, they should benefit from high-quality research that advances medical care for all. The curation of health data requires careful consideration of their downstream research uses and their many ethical implications, particularly when these data are used to develop health-care algorithms. Current frameworks of health data collection and distribution, whether from industry, academia, or government institutions, are imperfect and do not allow researchers to use the full potential of downstream analytical efforts. To take advantage of the wealth of data from clinical care, key stakeholders should come together to make health-care datasets increasingly accessible, inclusive, and representative, at the same time balancing the privacy and rights of the patients involved.

## *Panel*: Major commercial health datasets and vendors

### Optum (UnitedHealth Group)

Optum, a subsidiary of UnitedHealth, offers a database of administrative health insurance claims as part of its Clinformatics Data Mart. The database comprises deidentified medical, pharmacy, and laboratory data collected since 2000 across the USA.

### Merative MarketScan (formerly IBM MarketScan)

Merative MarketScan Research Databases comprise deidentified patient-level health data from privately and publicly insured Americans, including prescriptions, laboratory results, and electronic medical records. The dataset covers more than 273 million unique patients since 1995.

### Flatiron Enhanced Datamart

Flatiron, which was acquired by Roche in 2018, offers 22 datasets as part of its Enhanced Datamart, with a focus on clinical, genomics, and outcomes data related to oncology.

### IQVIA Real World Data

IQVIA, formed from the merger of IMS Health and Quintiles in 2016, offers an expansive portfolio of health data from electronic medical records, prescriptions, insurance claims, and other sources.

### Decision Resources Group Real World Evidence

Clarivate Real World Data offers data on over 300 million US patients, including over 120 million claims and electronic health records. These data cover demographics, laboratory results, diagnoses, and more.

### Bluehealth Intelligence (Blue Cross Blue Shield)

BlueHealth Intelligence offers a dataset of insurance claims with high geographic coverage and uniformity of structure for over 234 million unique patients.

### Symphony Integrated Dataverse

Symphony Health’s Integrated Dataverse connects longitudinal data from physicians, pharmacies, patients, and hospitals collected for 17 years from more than 317 million patients.

### Premier Healthcare Database

The Premier Healthcare Database includes hospital admissions data from more than 1000 US health-care providers and 231 million patients. Around three-quarters of the data are classified as originating from urban areas, with the remainder from rural regions.

### Cerner Real-World Data

Cerner Real-World Data offers a US national, deidentified, longitudinal patient dataset.

### PointClickCare Real World Data

PointClickCare offers more than 20 years of deidentified real world data from long-term and post-acute care, including nursing and assisted living facilities.

### Ontada Real World Data

Ontada is a data platform launched by McKesson, which offers patient demographics, diagnostics, treatments, insurance claims, and other data related to oncology care.

## Search strategy and selection criteria

For this Health Policy, we searched Google Scholar, PubMed, and Google for studies, commercial press releases, and reports published between Jan 1, 2011, and Dec 31, 2022, with the terms: “open health data”, “health data vendors”, “electronic health records”, “commercial health database”, “medical big data”, “real-world evidence”, “administrative claims data”, “biomedical reproducibility”, “biomedical generalizability”, “biomedical data accessibility”, and “biomedical data privacy”. We also included publications cited in the documents when relevant. We restricted the language of the studies, commercial press releases, and reports to those published in English. We included publications from searches if they presented a concept, principle, case study, or approach that was considered applicable either directly or indirectly to the use of commercial or non-commercial health datasets in biomedical research and health-care algorithms development.
